# Novel Mode of Treating Retention of Urine

**Published:** 1826-07

**Authors:** 


9. *Retention of Urine.* It would appear that surgery has come to such a pitch of perfection, that surgeons will be put to their wit's ends to invent difficulties. M. Brander seems to have set the example in the Quixotic path. In cases of retention of urine, when all other means fail, and an operation is indispensable, he proposes to *bore* the symphysis pubis, and come to the bladder in that direction, rather than above the pubes or through the rectum or perineum ! If this be not making difficulties where there are none, we know not what is. In puncturing the bladder above the pubes there is neither danger nor difficulty, and we would recommend this operation in preference to all others.—*N. Biblioth. Dec.*

